# Serotonergic Hyperactivity as a Potential Factor in Developmental, Acquired and Drug-Induced Synesthesia

**DOI:** 10.3389/fnhum.2013.00657

**Published:** 2013-10-21

**Authors:** Berit Brogaard

**Affiliations:** Department of Philosophy and Center for Neurodynamics, University of MissouriSt. Louis, MO, USA

**Keywords:** acquired synesthesia, autism spectrum disorder, drug-induced synaesthesia, left-hemisphere injuries, multisensory perception, savant syndrome, serotonin hypothesis, traumatic brain injury

## Abstract

Though synesthesia research has seen a huge growth in recent decades, and tremendous progress has been made in terms of understanding the mechanism and cause of synesthesia, we are still left mostly in the dark when it comes to the mechanistic commonalities (if any) among developmental, acquired and drug-induced synesthesia. We know that many forms of synesthesia involve aberrant structural or functional brain connectivity. Proposed mechanisms include direct projection and disinhibited feedback mechanisms, in which information from two otherwise structurally or functionally separate brain regions mix. We also know that synesthesia sometimes runs in families. However, it is unclear what causes its onset. Studies of psychedelic drugs, such as psilocybin, LSD and mescaline, reveal that exposure to these drugs can induce synesthesia. One neurotransmitter suspected to be central to the perceptual changes is serotonin. Excessive serotonin in the brain may cause many of the characteristics of psychedelic intoxication. Excessive serotonin levels may also play a role in synesthesia acquired after brain injury. In brain injury sudden cell death floods local brain regions with serotonin and glutamate. This neurotransmitter flooding could perhaps result in unusual feature binding. Finally, developmental synesthesia that occurs in individuals with autism may be a result of alterations in the serotonergic system, leading to a blockage of regular gating mechanisms. I conclude on these grounds that one commonality among at least some cases of acquired, developmental and drug-induced synesthesia may be the presence of excessive levels of serotonin, which increases the excitability and connectedness of sensory brain regions.

## INTRODUCTION

Synesthesia is an extraordinary way of perceiving the world, involving experiences of connections between seemingly unrelated sensations, images or thoughts (Baron-Cohen et al., 1987; Cytowic, 1989; Rich and Mattingley, 2002; Sagiv and Ward, 2006; Brogaard, 2012). For example, seeing the number 7 may lead to an experience of navy blue, hearing the word “bliss” may flood the mouth with the flavor of bread soaked in tomato soup and hearing the key of C# minor may elicit a bright purple spiral radiating from the center of the visual field. The trigger of the experience is called “the inducer,” whereas the additional experience to which it gives rise is called “the concurrent” (Grossenbacher and Lovelace, 2001). In visual synesthesia, the concurrent may be projected out into space and experienced as located in the visual scene outside the subject’s mind, or it may be merely imagistically or semantically associated with the inducer (Dixon et al., 2004).

The two key characteristics of synesthesia regardless of whether it is of the projector or associator type is that it involves an aberrant binding of features from different sensory or cognitive streams that are associated with atypical conscious experiences or thoughts and that these experiences or thoughts are automatic, that is, synesthetes cannot suppress the association between an inducer and its concurrent. Other characteristics of the condition are specific to the different forms. According to Grossenbacher and Lovelace (2001), there are three different types of synesthesia:

(1) Developmental, or genuine, synesthesia
(1) Acquired synesthesia
(1) Drug-induced synesthesia

Developmental synesthesia, the most common type, is a form of the condition that has persisted since birth or early childhood and that remains relatively stable and systematic over time: each inducer has a highly specific concurrent. (Baron-Cohen et al., 1987; Mattingley et al., 2001; Simner et al., 2006). It also tends to run in families (Baron-Cohen et al., 1996). For the most common forms of developmental synesthesia, the Synesthesia Battery, an automated online test, allows for rigorous testing of both the tightness of the synesthetic association and its stability and systematicity over time (www.synaesthete.org; Eagleman et al., 2007).

Acquired synesthesia is a form of the condition that emerges after brain injury or disease or artificial technologies like sensory substitution (Ward and Wright, 2012). It has been reported following stroke (Ro et al., 2007; Beauchamp and Ro, 2008; Thomas-Anterion et al., 2010; Schott, 2012), traumatic brain injury (Brogaard et al., 2012; Brogaard and Marlow, 2013), neuropathology involving the optic nerve and/or chiasm (Jacobs et al., 1981; Armel and Ramachandran, 1999; Afra et al., 2009), seizures (Jacome and Gumnit, 1979), migraine (Alstadhaug and Benjaminsen, 2010), post-hypnotic suggestion (Cohen Kadosh et al., 2009) and sensory substitution (Ward and Wright, 2012). Audio-visual synesthesia has been reported to be the most common acquired type (Afra et al., 2009). Like developmental synesthesia, acquired synesthesia tends to be automatic and systematic over time, though in some cases it only persists for a limited time period (Jacome and Gumnit, 1979; Lessell and Cohen, 1979; Afra et al., 2009). Experientally, acquired synesthesia may be indistinguishable from developmental synesthesia, though it is sometimes less inducer-specific, that is, the same concurrent may have several different inducers (Brogaard et al., 2012). Cases have also been reported in which the acquired experience is simpler than the developmental counterpart, often similar to light flashes (phosphenes) or pure color experiences (Afra et al., 2009).

Drug-induced synesthesia is a blending of sensory or cognitive streams that is experienced during exposure to a hallucinogen (psilocybin, LSD, mescaline, peyote; Shanon, 2002; Friedrichs, 2009; Sinke et al., 2012). Unlike the developmental and acquired varieties, the drug-induced form is usually limited to the most intense phases of intoxication, though in some cases it continues for weeks or months after exposure to the drug (Abraham, 1983; Ffytche, 2007). Experientally, drug-induced synesthesia can vary from simple color experiences to complex, surrealistic landscapes consisting of, for example, oddly shaped objects with multicolored contours or images with ornamental or kaleidoscopic compositions (Hintzen and Passie, 2010).

Though synesthesia research has seen a huge growth in recent decades, and tremendous progress has been made in terms of understanding the mechanism and cause of the condition, we are still left mostly in the dark when it comes to the mechanistic commonalities (if any) among developmental, acquired and drug-induced synesthesia. It is widely believed that most forms of the condition involve functional or structural aberrant brain connectivity. The proposed mechanisms include direct or indirect projection through increased structural connectivity (Ramachandran and Hubbard, 2001a, b; Hubbard et al., 2005; Rouw and Scholte, 2007; Jancke et al., 2009; Hanggi et al., 2011; Zamm et al., 2013), functionally driven disinhibited-feedback mechanisms (Grossenbacher and Lovelace, 2001; Dixon et al., 2006; Esterman et al., 2006; Neufeld et al., 2012), and mixed models (Hubbard, 2007; Ward, 2013). However, it is unclear what causes the onset of the condition and whether the different types of synesthesia have different causes.

One proposal by Brang and Ramachandran (2007) suggests serotonin (5-HT) as a causal factor. Their specific suggestion is that “serotonin S2a receptors are the ‘synesthesia receptors’ in the brain” (p. 903). In support of this hypothesis they list four pieces of evidence: (i) LSD produces synesthesia by selectively activating serotonin 5-HT2A receptors. (ii) Prozac (fluoxetine), a selective serotonin reuptake inhibitor that increases 5-HT1 receptor activity thereby inhibiting 5-HT2A, blocked synesthesia in two subjects. (iii) the anxiolytic drug Wellbutrin (bupropion), which presumably inhibits 5-HT2A receptor activity, temporarily abolished synesthesia in one subject, (iv) melatonin, a brain hormone derived from serotonin that can disinhibit 5-HT2A receptor activity, temporarily induced grapheme-color synesthesia in a subject with number-form synesthesia.

Thus, Brang and Ramachandran’s (2007) suggestion is that serotonin may be functionally implicated in generating synesthetic experience through 5-HT2A receptor activity. In the formulation of the hypothesis Brang and Ramachandran (2007) do not specify which of the 5-HT2A receptors in the brain cause synesthesia, whether the serotonin receptors that are “synesthesia receptors” are inhibitory or excitatory, whether serotonin could be structurally implicated in causing synesthesia through altered structural connectivity during brain development and whether the serotonin hypothesis may also explain cases of acquired synesthesia. My aim in this paper is to develop the serotonin hypothesis to tentatively answer these questions by looking at a wider range of evidence.

More specifically, my proposal is that excessive extracellular serotonin (5-HT) can be a trigger of persistent or transient synesthesia in all the groups through *excitatory* mechanisms. Though serotonin traditionally has been considered an inhibitory neurotransmitter, more recent evidence suggests a more complex picture according to which serotonin can function both as an inhibitory and an excitatory neurotransmitter. For example, serotonin helps reduce fear processing in the amygdala via GABA modulation but it exerts an excitatory effect on cortical brain activity when it binds to 5-HT2A serotonin receptors on layer V pyramidal neurons (Barkai and Hasselmo, 1994; Aghajanian and Marek, 1999).

As indicated by Brang and Ramachandran (2007), the suggestion that serotonergic activity may be a trigger of synesthesia has the greatest degree of evidential backing in the case of drug-induced synesthesia. It has been shown in several studies that psychedelic hallucinogens that function primarily as serotonin agonists, such as psilocybin, LSD and mescaline, often induce transient, often auditory-visual synesthesia, presumably through an alteration of functional brain connectivity. Though not all serotonin agonists elicit synesthetic experience, it is widely agreed that the mechanism of action for the class of serotonergic hallucinogens is through binding of serotonin to the 5-HT2A serotonin receptor (Presti and Nichols, 2004; González-Maeso et al., 2007).

Excessive serotonin levels may also play a role in synesthesia acquired after brain injury. Research has shown that necrosis following tissue damage leads to local neurotransmitter flooding caused by an excessive release of serotonin and glutamate (Busto et al., 1997; Hinzman et al., 2010), a phenomenon that can have long-range consequences even when the injury is minor. This flooding appears to lead to increased functional or structural interconnectedness among different brain regions in some individuals, and in some cases this increased connectivity may be a cause of synesthesia.

Developmental synesthesia has been reported as a condition in autism-spectrum disorders (Ornitz et al., 1978; Grandin, 1995; Kemner et al., 1995; Harrison and Hare, 2004; Asher et al., 2009; Baron-Cohen et al., 2009), and is believed to be more frequently occurring in individuals with autism compared to the general population (Cytowic, 1989). It is suggested below that synesthesia accompanying autism may be a serotonergic condition, resulting from excessive serotonin levels in early childhood that could possibly lead to decreased extracellular levels of serotonin in one hemisphere and compensatory increased levels in the other hemisphere. One piece of evidence for this comes from PET scans of people with high-functioning autism which have revealed that in the majority of cases serotonin synthesis is suppressed in the left hemisphere and increased in the right hemisphere, though in some cases it is reversed (Chandana et al., 2005). Recent genetic studies furthermore suggest a genetic link between autism spectrum disorder and developmental synesthesia in non-autistic individuals (Asher et al., 2009). Whether there is a mechanistic link between autism and cases of developmental synesthesia in non-autistic individuals remains to be established.

In what follows I will provide detailed evidence for the hypothesis that one commonality among at least some cases of acquired, developmental and drug-induced synesthesia is the presence of excessive levels of serotonin or serotonin-agonists, which increase the excitability and connectedness of sensory brain regions through 5-HT2A receptors in cortical neurons.

## ACQUIRED SYNESTHESIA

The acquired form of synesthesia usually emerges subsequent to traumatic brain injury or neuropathologic insult to the brain (Beauchamp and Ro, 2008; Afra et al., 2009; Brogaard et al., 2012). Several studies have hypothesized that these acquired synesthesias occur from plasticity of the sensory systems resulting in increased connectivity (Ro et al., 2007; Beauchamp and Ro, 2008). This theory has additionally been studied in at least one case of acquired synesthesia (Ro et al., 2012). Ro et al. (2012) found that connections between the auditory and somatosensory cortices in healthy controls were strengthened in a subject with auditory-tactile synesthesia acquired after a right ventrolateral thalamic lesion that deprived her somatosensory cortex of normal somatosensory input.

It remains largely unknown how a brain lesion may give rise to the plastic changes that lead to the increased connectivity. One possibility is that it is caused by increased neurotransmitter activity in cortical regions adjacent to the affected site. This increase is believed to contribute to the pathophysiology and neurological dysfunction after traumatic brain injury (Busto et al., 1997; Bullock et al., 1998; Robertson et al., 2001; Werner and Engelhard, 2007; Hinzman et al., 2010). In traumatic brain injury tissue damage makes the cells shift to anaerobic glycolysis, resulting in an accumulation of lactic acid. The anaerobic metabolism cannot by itself maintain the energy levels, so ATP-stores are depleted and the membrane ion-pumps, which depend on ATP, fail. This leads to membrane degradation of vascular and cellular structures and necrotic or programmed cell death (apoptosis), resulting in excessive release of excitatory neurotransmitters, particularly serotonin and glutamate (Busto et al., 1997; Hinzman et al., 2010). This excess in extracellular serotonin and glutamate availability affects neurons and astrocytes and results in over-stimulation of serotonin and glutamate receptors. It is believed that this over-stimulation effect can happen even in mild traumatic brain injury, as the release of neurotransmitters is vast even with minor lesions (Konrad et al., 2011; Perez-Polo et al., 2013). Other types of brain injury that have been reported to trigger synesthesia, such as stroke (Ro et al., 2007; Beauchamp and Ro, 2008; Afra et al., 2009; Thomas-Anterion et al., 2010; Schott, 2012), also cause neurotransmitter flooding after necrosis. It has been found, for example, that even a brief ischemic stroke may trigger complex biochemical events that lead to progressive apoptotic and necrotic neuronal cell death (Yuan, 2009).

The increased levels of serotonin and glutamate immediately following brain injury do not normally stay elevated for very long (Eysel et al., 1999). Eysel et al. (1999) induced 1.5 to 2 mm lesions in the striate cortex of cats using surface photocoagulation or ibotenic acid injections. Single cell measurements revealed that activity was decreased at the border of the lesion and increased in a ring around the lesion during the first days to weeks following the induction but it returned to normal after about a month (Eysel et al., 1999). Despite the fact that serotonin does not seem to stay elevated for more than one month following a brain lesion, there is suggestive evidence that the temporary elevation may suffice for creating long-lasting functional, and possibly also structural, changes (Giza and Prins, 2006; Konrad et al., 2011; Perez-Polo et al., 2013). It has furthermore been reported that initial increases in neurotransmitter levels following brain injury or disease may be followed by down-regulation of receptors in ipsilateral brain regions (Giza and Prins, 2006).

One hypothesis for how brain injury or disease may cause synesthesia, then, is that the initially elevated neurotransmitter levels down-regulate serotonin receptors in neural regions in the ipsilateral hemisphere, leading to decreased serotonin levels. Individuals who acquire synesthesia also sometimes develop autistic traits and savant-like abilities of the sort seen in 10 percent of autistic individuals (Baron-Cohen et al., 2007; Treffert, 2009; Brang and Ramachandran, 2011; Rogowska, 2011; Brogaard et al., 2012; Schott, 2012; Ward, 2013). Emerging savant skills have also been reported in many other cases of central nervous system injury or disease later in life (Fay, 1987; Dorman, 1991; Miller et al., 1998, 2000; Lythgoe et al., 2005; Tammet, 2006; Treffert, 2006, 2009; Snyder, 2009). As is known from studies of autistic savants, down-regulation of the serotonergic system in one hemisphere may result in an upregulation of the serotonergic system in contralateral brain regions (DeLong, 1999; Takeuchi et al., 2012), which might explain the development of savant skill and synesthesia following brain injury (Treffert, 2009). A second hypothesis is that the elevation in serotonin and glutamate levels in the days or weeks after brain injury can trigger disinhibited feedback or a structural binding of features through serotonergic hyperactivity in sensory neurons or neurons in parietal cortex involved in mental imagery. The latter have been found to be plausible neural correlates in at least one case of acquired synesthesia (Brogaard et al., 2012).

The second hypothesis is more plausible than the first when synesthesia is acquired after brain injury. It would explain the increased ipsilateral connectivity found in at least one case of acquired synesthesia (Ro et al., 2012). It gains further support from the fact that the onset of synesthesia following brain injury has been reported to occur shortly after the injury rather than months later, which indicates that it is the initial neurotransmitter flooding that causes the onset. Furthermore, if the first hypothesis were correct, then the affected region would have to be crucially implicated if the down-regulation triggers an upregulation in other areas, an assumption that cannot be confirmed at the present time. The first hypothesis may have a greater degree of support for synesthesia acquired after frontotemporal dementia. Cell death in frontotemporal dementia does not normally occur via necrosis that leads to neurotransmitter flooding. However, it is well known that patients with this condition have deficits in serotonergic and dopaminergic signal-transmission. These deficits may account for some of the cognitive and behavioral impairments of the disease. As prefrontal areas are known to exert inhibitory control over other brain regions, decreased activity in these neural regions could lead to a disinhibitory enhancement of neural activity and connectivity in unaffected cortical regions (DeLong, 1999; Treffert, 2009), which could also explain why this type of dementia frequently is reported as a cause of savant skills (Miller et al., 2000). Regardless of whether the synesthesia is acquired after brain injury or dementia, neuroimaging might help reveal whether the unusual binding is functional or structural. For example, if it is functional, we should expect to find increased connectivity in functional connectivity analyses on fMRI data.

The above considerations raise the question of why only a small fraction of brain injury patients acquire synesthesia-like experiences. One possible answer is that individual differences originate in variance in the degree of plasticity of the subject’s brain prior to the incident. Research on brain injury in children has shown that alterations in neurotransmission during the critical period when a brain region is most plastic can promote outgrowth of abnormal neural connections (Giza and Prins, 2006). Since it is likely that there are significant individual differences in the plasticity of the mature brain, it is possible that some subjects are more susceptible to the formation of new neural connections than others. Individual differences are no doubt also grounded in variance in the location of the injury. Synesthesia-like experiences may be much more likely to develop if sensory regions or neural areas implicated in mental imagery are affected.

Given the broad range of conditions that can trigger acquired synesthesia it is unlikely that there is a single mechanism underlying all cases. Some cases appear to be quite similar in persistence and phenomenology to well-known forms of developmental synesthesia and are likely to share a neurological basis with some of these varieties of synesthesia. For example, autistic savant Daniel Tammet describes in his book *Born on a Blue Day* that he acquired synesthesia after childhood seizures. Though his grapheme-color synesthesia appears unusually rich, it may have neurological underpinnings akin to more typical developmental forms. Other forms of acquired synesthesia appear to be transient and may well be a more direct product of excitatory neural activity, similar in many respects to synesthetic experience occurring under the influence of hallucinogens (see below). An example would be synesthesia experienced during occipital and temporal lobe seizures, which can lead to brief experiences quite similar to psychedelic experiences (Devinsky and Luciano, 1991; Cytowic, 1997; Sacks, 2012). Some forms of acquired synesthesia could be undergird by a different mechanism not typically found after drug intoxication or aberrant neural development. In cases in which synesthesia and savant syndrome are acquired in the same incident, the two conditions may well be triggered in similar ways. This could be the case for Tammet, who reports having synesthesia matching not only digits but also the product of digits. His synesthesia could possibly be an imagistic manifestation of number processing in the parietal cortex. One observation that supports this hypothesis is that the lack of increased activity in the visual cortex in response to synesthetic tasks found in an imaging study comparing brain activity in Tammet and controls (Bor et al., 2007). But more research needs to be done to settle these questions. In most cases of acquired synesthesia the research component has taken place several years after the onset of the condition. If we could perform the research (e.g., neuroimagining studies) closer to the onset, we might be able to determine whether serotonergic hyperactivity in sensory neurons is implicated in the sudden, unusual binding of features.

## DRUG-INDUCED SYNESTHESIA

Drug-induced synesthesia is a blending of perceptual or cognitive streams that emerges in subjects under the influence of psychedelic hallucinogens, psychoactive substances that alter perception, mood, and a variety of cognitive processes. A myriad of first-person reports indicate that synesthesia occurs during psychedelic intoxication. Some reports suggest an altered perception of the world that blends normally distinct senses. Reports of colored music are particularly frequent (Shanon, 2002, 2003; Sinke et al., 2012). In other cases external objects appear to the perceiver as having an unusual wealth of colors, textures and shapes that undergo rapid changes. Subjects report seeing melting windows, breathing walls and spiraling geometrical figures crawling over the surfaces of objects. Reflecting on a DMT session one subject, described by Cott and Rock (2008), reported that “The room erupted in incredible neon colors, and dissolving into the most elaborate incredibly detailed fractal patterns that I have ever seen.” The authors characterize this as a hallucination, and it is admittedly difficult to distinguish synesthesia and hallucinations, particularly because some forms of synesthesia probably are best characterized as hallucinations (Sagiv et al., 2011). A crucial difference seems to be that hallucinations *proper* do not have a phenomenally apparent inducer, whereas synesthesia does. Another difference is that most forms of synesthesia are experienced as endogenous images or representations (i.e., associator synesthesia, Dixon et al., 2004). Yet another difference is that synesthetes know that the concurrent experience is not a veridical perception, whereas hallucinations tend to evoke the experience of the veridicality of the perception (Terhune and Cohen Kadosh, 2012).

It is by now fairly well established that two major classes of psychedelic hallucinogens, the indoleamines (e.g., LSD and psilocybin) and the phenethylamines (e.g., mescaline), are potent partial agonists at serotonin 5-HT1A/2A/2C receptors, with 5-HT2A receptor activation directly correlated with hallucinogenic activity (Glennon, 1990; Vollenweider et al., 1998; Nichols, 2004; Presti and Nichols, 2004; though see e.g., Previc, 2011 for a different perspective). Though the mechanism of action varies for different hallucinogens, it is believed that 5-HT2A receptor activation of cortical neurons is responsible for mediating the signaling pattern and behavioral response to hallucinogens (Presti and Nichols, 2004; González-Maeso et al., 2007). However, the activation of the cortical serotonergic system does not fully explain the perceptual effects of psychedelic drugs, as not all 5-HT2A agonists (or partial agonists) have an excitatory mechanism of action and not all 5-HT2A agonists have psychedelic effects (e.g., methysergide). So, this raises the question of what other factors need to be present for drug-induced hallucinations to occur.

A promising suggestion for how the hallucinatory effects occur is that hallucinogens activate layer V pyramidal neurons in the cortex, which engage in gating functions in communication between the cortex and subcortical brain regions (Barkai and Hasselmo, 1994). When a hallucinogen binds to the 5-HT2A receptor, this gives rise to an excitatory response (Scruggs et al., 2003; Nichols, 2004). Recent research suggests that co-transmission of glutamate and monoamines is a frequent occurrence in the central nervous system (Trudeau, 2004; Ciranna, 2006). The 5-HT2A receptors, specifically, have been found to increase glutamate release (Ceglia et al., 2004; Torres-Escalante et al., 2004). There is furthermore evidence suggesting that the hallucinogen psilocybin targets a cortical receptor complex that forms when the glutamate mGluR2 receptor interacts with the serotonin 5-HT2A receptor (González-Maeso et al., 2008). Increased release of glutamate in response to hallucinogen administration should enhance cortical metabolic activity, a finding that has been confirmed by Vollenweider et al. (1997). Perceptual changes have been found to correlate with increased metabolic activity in the frontomedial and frontolateral cortices, anterior cingulate, and temporomedial cortex (Vollenweider et al., 1997). There is also some evidence from EEG studies that activation of 5-HT2A receptors increases the excitability of cortical sensory networks by modulating alpha oscillations (8–12 Hz; Kometer et al., 2013). Brain waves in the alpha frequency range have been shown to regulate the excitability levels of cortical sensory networks through inhibition (Klimesch, 2011). The increased excitatory action of serotonin and glutamate in sensory regions might explain why hallucinogens mimic aspects of psychosis during intoxication (Aghajanian and Marek, 1999; Pralong et al., 2002; Nichols, 2004; González-Maeso et al., 2008).

**FIGURE 1:**
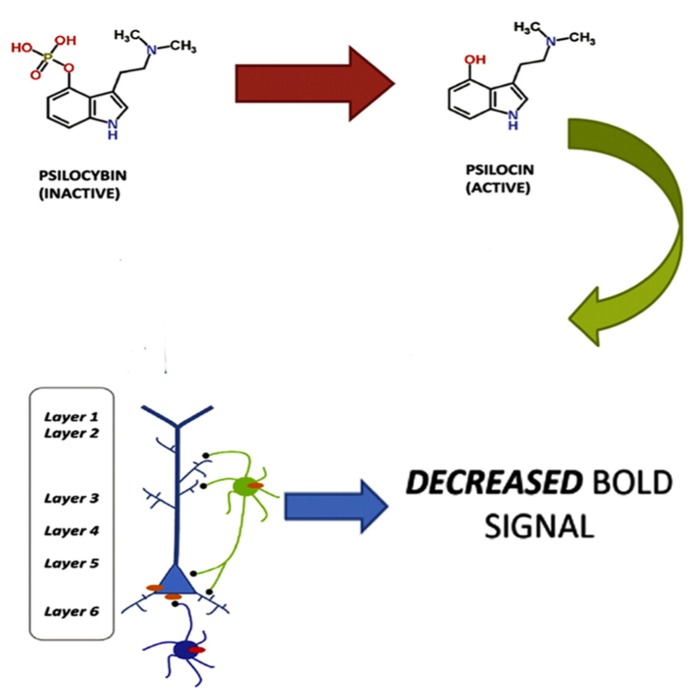
**Psilocybin, which is inactive, is metabolized to the active ingredient psilocin.** Psilocin then triggers a 5-HT2A excitatory response in layer V pyramidal neurons and an inhibitory response in GABAergic interneurons. The inhibitory response produces a decreased BOLD response. Adapted from Lee, H.-M. and Roth, B. L. (2012). Hallucinogen actions on human brain revealed. PNAS 109, 6: 1820–1821.

The view that the hallucinatory effects of hallucinogens are primarily a result of enhanced neural activity may appear inconsistent with a recent fMRI study showing that psilocybin causes decreased activity in the ACC/medial prefrontal cortex and a significant decrease in the positive coupling between the medial prefrontal cortex and the posterior cingulate cortex, and that these findings were correlated with subjective effects (Carhart-Harris et al., 2012). As Lee and Roth (2012) point out, however, these results are consistent with the observation that 5-HT2A receptors are found on both layer V glutamatergic neurons and GABAergic interneurons. Furthermore, there is also a direct activation of GABAergic interneurons through the synapses of pyramidal cells onto the interneurons (Markram et al., 2004). So, a large excitatory response in a pyramidal neuron will lead to a large inhibitory response in the interneuron. Lee and Roth (2012) propose that the effects of psilocybin could be due to both excitatory (e.g., pyramidal) and inhibitory (e.g., GABAergic interneuronal) neuronal circuits and that it may be the effects on the inhibitory neuronal circuits that gave rise to the measured BOLD response (see **Figure 1**).

To my knowledge, no model of how hallucinogens trigger synesthesia has yet been proposed. One natural proposal would be that the mechanisms in developmental and drug-induced synesthesia are similar. There is, however, some reason to doubt this suggestion. The phenomenological differences between developmental and drug-induced synesthesia are quite striking (see Sinke et al., 2012 for a review). As experiences causally supervene on neurological processes, experiences that are significantly different in their phenomenology are bound to have significantly different neurological underpinnings. So, we should expect some differences in the underlying mechanisms.

**FIGURE 2:**
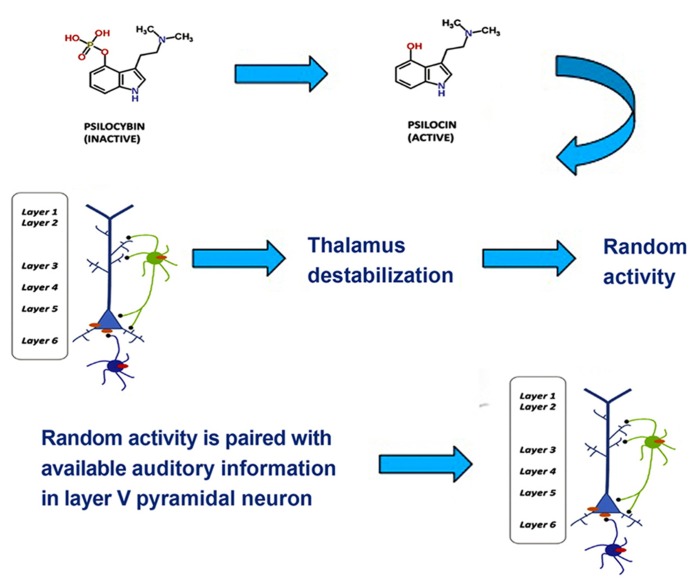
**Psilocybin, which is inactive, is metabolized to the active ingredient psilocin.** Psilocin then triggers a 5-HT2A excitability response in layer V pyramidal neurons and an inhibitory response in GABAergic interneurons. This leads to thalamic destabilization, which triggers random thalamic activity. The occipitally processed random activity is paired with available auditory information in layer V pyramidal neurons, which yields synesthetic experience. Adapted from **Figure 1**.

One tentative suggestion is that drug-induced synesthesia, like hallucinations, originates in the hyperactivity of layer V pyramidal cells resulting from the binding of hallucinogens to 5-HT2A receptors in the cells’ dendrites, which then increases local glutamate levels. Layer V pyramidal cells bind multisensory information through feedback loops that synchronize oscillatory neural responses (Guillery and Harting, 2003). In the visual and the auditory cortices layer V neurons form feedback loops with local neurons as well as neurons in the thalamus and prefrontal cortex. Projections to thalamus play a role in discriminating among incoming information and integrating information from different sensory channels, whereas projections to the prefrontal cortex play a role in higher-order processes and the generation of a conscious representation. In normal multisensory perception, low-level multisensory binding of incoming signals from visual and auditory channels occurs spontaneously in the auditory cortex via thalamocortical feedback loops, when the spatial and temporal attributes of incoming signals match (Schroeder and Foxe, 2005). Excessive excitatory activity in layer V pyramidal neurons, however, results in a destabilization of layer V projections to the thalamus through GABAergic neuronal circuits (Kim and McCormick, 1998; Markram et al., 2004). This has a number of consequences, such as decreased attentional discrimination among incoming stimuli, allowing more information to flood the sensory cortices, a loss of stimulus-specific inhibition, an resultant increase of random (or environmentally under-constrained) activity in the thalamus, and a disruption of low-level, spontaneous integration of multisensory stimuli on the basis of *actually* matching spatial and temporal attributes. The disruption of low-level integration mechanisms can result in incongruent experiences, such as hearing an object hit the floor prior to seeing it fall. Another result of this disruption of low-level integration may be a coupling of stimuli that do not belong together. A common synesthetic experience during hallucinogen intoxication is colored, geometrical grids, matrices or fractals induced by music (see Sinke et al., 2012 for a review). These types of visual experience also frequently occur without an inducer, probably as a result of random activity in the thalamus (Behrendt and Young, 2004; Sagiv et al., 2011). One possible mechanism for drug-induced synesthesia, then, is that the brain assumes that an experience that results from occipital processing of random thalamic activity matches auditory stimuli, leading to an unusual low-level binding in the auditory cortex (see **Figure 2**). As a result of this aberrant binding, the two inputs may be experienced as an inducer-concurrent pair, for example colored, geometrical music.

The proposed mechanism leads to the testable prediction that drug-induced synesthesia will not usually be systematic. In developmental cases sound-color synesthesia is systematic in the sense that very specific sounds normally trigger very specific colors. Given the aberrant binding proposed, however, it appears that drug-induced sound-color synesthesia will not tend to be systematic because random activity will be paired with available auditory information. So, we should expect that the same sound may have many different colors and shapes.

There are only few reports of drug-induced synesthesia that endures post-exposure, though there is evidence that other practices that can induce altered states of consciousness, such as meditation and posthypnotic suggestion, frequently cause enduring synesthesia (Walsh, 2005; Cohen Kadosh et al., 2009). The suggested mechanism may help explain why drug-induced synesthesia is seldom persistent over time. The hypothesis proposed is that occipitally processed random activity is coupled with available auditory information during drug-induction. The random activity does not persist in the same magnitude after drug exposure, which may explain why the synesthetic experiences and hallucinations tend to subside. But what further decreases the chance that the synesthesia persists would be the lack of a one-one mapping or even a many-one mapping from inducer to concurrent, which we might expect will make the unusual synesthetic binding less likely to be retained as a neural connection.

The proposed mechanism of hyperexcitability followed by disruption may appear to be inconsistent with a related finding for developmental synesthesia. It is believed that enhanced cortical excitability at an early developmental stage might contribute to atypical grapheme-color binding, even though it does not appear to be a direct cause of synesthesia at later stages (Asher et al., 2009; Tomson et al., 2011). A recent study reported data that were consistent with the idea that at later stages the hyperexcitability can hinder synesthesia by producing excess noise in the visual cortex and thereby reducing conscious awareness of the atypical binding of features (Terhune et al., 2011). However, I think that this suggestion can be reconciled with the proposed mechanism for drug-induced synesthesia. It is plausible that hyperexcitability may no longer play a functional role at later stages of developmental synesthesia (Terhune et al., 2011). Hallucinogenic serotonin agonists, however, appear to introduce a significant amount of hyperexcitability that can offset normal binding by disrupting low-level integration mechanisms. It is possible that some varieties of developmental synesthesia form in early childhood in a way similar to the way drug-induced synesthesia is here hypothesized to form temporarily later in life, but there would be certain differences: developmental synesthesia is systematic so if synesthesia develops in childhood as a result of a disruption of low-level integration, it is not a result of random activity in the thalamus. Furthermore, the presence of the hyperexcitability during brain development must somehow make it more likely that the unusual binding persists over time than when the hyperexcitability is drug-induced.

A version of the proposed model also appears consistent with the findings of Cohen Kadosh et al. (2009). The team induced strong projector grapheme-color synesthesia using posthypnotic suggestion in a group of highly suggestible college students. In most cases the synesthesia was found to endure and to display the phenomenal characteristics of developmental grapheme-color synesthesia. Cohen Kadosh et al. (2009) provide evidence suggesting that the induced synesthesia resulted from posthypnotically triggered disinhibited feedback. However, their results seem equally consistent with a model that proposes disruption of low-level integration. One shortcoming of both mechanistic proposals for synesthesia induced post hypnosis is that they do not explain why the condition tends to endure much longer than drug-induced synesthesia.

A further interesting question is whether the above model could be adapted to explain acquired synesthesia in which necrosis leads to neurotransmitter flooding and elevated local serotonin levels. Though we do not have enough data on acquired synesthesia to make any firm conclusions at this point, it remains a possibility that some forms of developmental synesthesia are acquired in childhood in the same way as the persistent cases of acquired synesthesia, whereas temporarily acquired synesthesia is mechanistically akin to drug-induced synesthesia. If this is correct, then we should expect drug-induced synesthesia and temporarily acquired synesthesia to both lack systematicity, as they would result from pairing features with random information. We should furthermore expect persisting forms of acquired synesthesia to have the same patterns of permanence and systematicity as at least some forms of developmental synesthesia.

## DEVELOPMENTAL SYNESTHESIA IN AUTISTIC AND NON-AUTISTIC INDIVIDUALS

Though it is difficult to say whether alterations in the serotonergic system play a role in the development of the most common forms developmental synesthesia, such as week-color and grapheme-color, developmental synesthesia seen in autism spectrum disorder is plausibly triggered by unilateral changes in the serotonergic system (Milner, 1973; Chugani et al., 1997; DeLong, 1999). There is not yet any solid evidence that autism and synesthesia are mechanistically related but there appears to be a statistical correlation as well as a possible genetic connection between the two conditions: synesthesia as well as sensory and perceptual abnormalities, such as hyper-responsiveness to sensory stimulation, are often reported as significant symptoms in autism-spectrum disorders (Ornitz et al., 1978; Grandin, 1995; Kemner et al., 1995; Harrison and Hare, 2004; Asher et al., 2009; Baron-Cohen et al., 2009). Cytowic (1989) states that 15 percent of people with autism experience synesthesia, which would be significant compared to the 4.4 percent of the general population (Simner et al., 2006). However, a systematic population study of the correlation between autism and synesthesia has not yet been completed. About 10 percent of individuals with autism also have savant syndrome, and all three conditions have frequently been reported to occur together (Baron-Cohen et al., 2007; Bor et al., 2007; Rothen et al., 2012). Recently, a genetic link between synesthesia and autism was suggested (Asher et al., 2009). Despite the lack of solid evidence for a mechanistic connection between autism and synesthesia, the existing statistical and genetic correlations give us reason to explore how serotonin may be related to synesthesia in autistic individuals. I will look at the evidence for the serotonin hypothesis for autism and then propose some testable predictions about how serotonin may give rise to synesthesia in autistic individuals.

The evidence that serotonin plays a crucial role in autism is overwhelming. About 30 percent of autistic individuals have a 25 to 70 percent increase in blood levels of serotonin, also known as hyperserotonemia (Schain and Freedman, 1961; Chugani et al., 1997, 1999; Veenstra-VanderWeele et al., 2012). Hyperserotonemia has also been found to a similar degree in first-degree healthy relatives (Leboyer et al., 1999). As serotonin cannot normally cross the blood-brain barrier in adults, high blood levels of serotonin are not necessarily a good indicator of high extracellular serotonin in the brain. High blood levels of serotonin, however, may indicate brain levels of serotonin in young children, as the blood-brain barrier is not fully developed until the age of two. Higher rates of autism have also been found in children exposed in utero to drugs that increase 5-HT levels, such as cocaine (Kramer et al., 1994). The high levels of serotonin in young children can negatively affect the development of serotonin neurons through negative feedback. As serotonin neurons develop and the extracellular levels of the neurotransmitter increase, growth of serotonin neurons is normally curtailed through a negative feedback mechanism, leading to a loss of serotonin terminals (DeLong, 1999; Whitaker-Azmitia, 2005). This decrease in serotonin terminal development has also been found in animal studies administering monoamine oxidase A and B inhibitors or serotonin reuptake inhibitors during gestation (Whitaker-Azmitia et al., 1994).

Several PET imaging studies have suggested that autism may be a lateralized syndrome, with decreased serotonin release from raphe terminals in one hemisphere (typically the left) and elevated serotonin release in the contralateral hemisphere (Chugani et al., 1997, 1999; Chandana et al., 2005). Significantly increased language impairment was found in subjects with decreased serotonin synthesis in the left hemisphere compared to individuals with right-hemisphere abnormalities and those without cortical asymmetry. Further evidence for the lateralization theory comes from studies indicating functional improvement with selective serotonin reuptake inhibitors (SSRIs), such fluoxetine (DeLong, 1999). SSRIs block serotonin transporters, preventing extracellular serotonin from being transported back into the cell. When children with autism are treated with SSRIs, the symptoms they share in common with individuals with major depressive disorder and anxiety disorders drastically improve. The children’s range of interests broaden, their perceptual experiences normalize, their memory and cognitive functions improve and anxiety and phobias become less prominent. Depleting brain serotonin in a tryptophan depletion paradigm, on the other hand, results in a worsening of the mood-related symptoms of autism as well as behaviors such as whirling, flapping, rocking and pacing (McDougle et al., 1996), though it does not affect the other symptoms of autism.

One possible explanation of the asymmetry is that the early serotonin depletion in the dominant left hemisphere leads to overcompensation in the right hemisphere. It has been reported that a decrease in extracellular serotonin over time may lead to an excessive spread of thalamocortical axon branches, resulting in lower information-transmission and structural changes in affected cortical regions as well as underdeveloped long-range connections between different brain areas (DeLong, 1999; Mueller et al., 2013). When the decrease in serotonin is left-lateralized, this leads to a general hypoexcitability of the left hemisphere and a wider right parietooccipital region compared to individuals with mental retardation or miscellaneous neurological disorders (Hier et al., 1979). The reversal of the asymmetry may be the result of initial right-hemisphere dominance, as indicated by a higher incidence of left-handedness and improved language skills in autistic individuals with decreased serotonin synthesis in the right hemisphere (Chandana et al., 2005). These results may explain why savant syndrome occurs in ten percent of autistic individuals. The leading hypothesis is that savant syndrome is caused by a lesion or birth defect in one hemisphere that results in overcompensation by the other hemisphere (Pesenti et al., 2001; Snyder et al., 2003; Young et al., 2004; Treffert, 2009; Loui et al., 2011).

A hemispheric effect does not provide clear evidence for a role of serotonin in synesthesia, as not all individuals with autism have synesthesia. But the expected high frequency of synesthesia in autistic individuals together with the lateralization hypothesis point to the possibility that increased extracellular levels of serotonin in the autistic brain may be a causal influence on the genesis of synesthesia in individuals with this disorder. There appears to be two ways in which serotonin could be implicated in synesthesia in autistic individuals. One possibility is that the high serotonin levels in very young children with autism sometimes trigger altered multisensory processing. Another possibility is that compensatory high serotonin levels in the contralesional hemisphere sometimes cause unusual feature binding. If the former hypothesis is correct, then we should expect to find evidence of synesthesia in autistic individuals at a very young age.

There are multiple ways that elevated serotonin levels may lead to synesthesia in autistic individuals. If the onset of synesthesia in autism occurs at an early age, it could be the result of serotonin-triggered hyperactivity in glutamatergic neurons in layer V and resulting destabilization of thalamic connections. If this hypothesis is correct for synesthesia in autistic individuals, then we should expect that the synesthetic connections can persist only in the form of tight memory connections after the subsequent loss of serotonin-terminals in large areas of the brain. Accordingly, on the assumption that projector synesthesia is not normally mnemonic, we should not expect to find a significant number of projector synesthetes among autistic individuals. Another way synesthetic connections could form would be through the early excessive formation of neural pathways. Persisting synesthesia in this case would require that the early formed structural connections could survive the extensive pruning that appears to take place when serotonin-terminals are lost. If the onset of the synesthesia does not occur at an early age, structural or functional synesthetic connections could still form in the spared right-hemisphere regions that are also believed to be responsible for the savant skills founds in 10 percent of autistic individuals. Structural connectivity mechanisms have been proposed for standard cases of grapheme-color synesthesia, suggesting unusual connectivity between the color area and the fusiform gyrus (Ramachandran and Hubbard, 2001a, b; Hubbard et al., 2005). Enhanced anatomical connectivity near the fusiform gyrus confirming this hypothesis has been reported for grapheme-color synesthesia (Rouw and Scholte, 2007; Jancke et al., 2009; Hanggi et al., 2011) and sound-color synesthesia (Zamm et al., 2013). Whether a structural connectivity mechanism also underlies synesthesia in autism could fairly easily be tested by using a DTI paradigm to look at whether there are similar patterns of localized hyper-cortical-connectivity in autistic individuals with synesthesia. If the onset of the synesthesia does not occur at an early age, there is also the possibility that it is the result of impaired higher-order multisensory integration associated with decreased functional and structural connectivity (Mueller et al., 2013). This would make developmental synesthesia in autism very different from other cases, which appear to be a result of increased structural or functional connectivity. There is, however, a fairly strong reason against this mechanism as explanatory of synesthesia in autistic individuals. Impaired multisensory integration in autism amounts to a failure to associate two high-level sensory input that neurotypical individuals would associate (Mueller et al., 2013), not a persistent success in associating two input that neurotypical individuals do not associate.

Whether a hyperactive serotonergic system can contribute to developmental synesthesia in non-autistic individuals is unknown. As autism is partly defined by sensory processing deficits, the emergence of synesthesia in autism could be a consequence of other sensory alterations and originate from a unique set of mechanisms distinct from those present in other cases of developmental synesthesia. But some data points indicate that serotonin may be mechanistically involved in producing synesthetic experience through altered functional connectivity. As Brang and Ramachandran (2007) observed, there is some reported pharmacological evidence suggesting that developmental synesthesia in non-autistic individuals could sometimes be a serotonergic condition. Pharmacological evidence for the serotonin hypothesis was also reported by Cytowic (1989). He describes a patient with life-long synesthesia who developed epilepsy as an adult and subsequently experienced less vivid synesthetic experiences when treated with the anti-epileptic drug Tegretol (carbamazepine), which is known to increase extracellular levels of serotonin (Cytowic, 1989, p. 174). The reports from Cytowic (1989) and Brang and Ramachandran (2007) may seem to provide evidence against serotonin triggering synesthesia via excitatory activity, as the increased serotonin levels apparently inhibited synesthesia. However, both fluoxetine and carbamazepine have been shown to significantly increase GABA and reduce glutamate levels, which would block the excitatory effects of serotonin in cortical areas (Kamal, 2010). So, these data suggest that serotonin may be functionally involved in generating synesthetic experience either through a disinhibited feedback mechanism or by making unusual structural binding available for conscious processing. The pharmacological evidence thus lends some support to a disinhibited feedback mechanism, which suggests that synesthesia is not a result of altered structural connectivity but arises from altered functional feedback connections. This type of mechanism has received prior support from psychophysical and neuromagining studies of non-autistic synesthetes (Grossenbacher and Lovelace, 2001; Dixon et al., 2006; Esterman et al., 2006; Neufeld et al., 2012). A recent imaging study of 14 auditory-visual non-autistic synesthetes, for example, found increased functional connectivity of the left inferior parietal cortex with the left primary auditory and right primary visual cortex (Neufeld et al., 2012), suggesting that the aberrant synesthetic binding takes place in parietal cortex. There is also suggestive evidence of mixed mechanisms. For example, some forms of grapheme-color synesthesia appear to involve enhanced visual memory associations with hyper-reinstantiation in the visual cortex (see Brogaard, 2013 and Brogaard et al., 2013 for reviews).

Drawing on evidence that the 5-HT2A receptor may be involved in generating synesthetic experience, Brang and Ramachandran (2007) suggest that synesthesia might occur from overexpression of the 5-HT2A receptor gene on chromosome 13. A whole-genome linkage scan and a family-linkage analysis in a sample of 43 multiplex families with auditory-visual synesthesia did not confirm this hypothesis (Asher et al., 2009). Instead the study suggested that synesthesia may be traceable to a region on chromosome 2 (2q24.1) that has been implicated in autism (Newbury et al., 2009), indicating that there may be genetic link between developmental synesthesia and autism.

Other evidence gives some credit to the hypothesis that synesthesia in non-autistic individuals could be related in terms of brain structure to autism and savant syndrome and hence that a particular brain structure may underlie all of these conditions. Population studies suggest that there may be a higher incidence of synesthesia among people with creative talent (Domino, 1989; Mulvenna and Walsh, 2005; Simner et al., 2006; Rothen and Meier, 2010). Conversely, some synesthetes appear to have greater cognitive and memory capacities specific to the concurrent of the individual’s synesthesia compared to the general population (Yaro and Ward, 2007; Ward et al., 2008; Banissy et al., 2009; Rothen et al., 2012). The possible association between synesthesia and cognitive talent might suggest that synesthetes without autism have serotonin-induced hyperconnected neural networks without the down-regulated neural regions found in people with autism. Recent neuroimaging studies further point to enhanced functional connectivity or increased gray matter density in synesthesia. A recent functional MRI study found increased intrinsic network connectivity in 12 grapheme-color synesthetes that reflected the strength of their synesthetic experiences (Dovern et al., 2012). Wiess and Fink (2009) further reported greater gray matter volume in the left intraparietal sulcus and right fusiform gyrus in 18 synesthetes compared to 18 controls. Several DTI studies have confirmed increased white matter connections in the superior parietal cortex, right inferior temporal cortex and frontal regions in grapheme-color synesthesia (Rouw and Scholte, 2007; Jancke et al., 2009, though at more liberal thresholds; Hanggi et al., 2011) and sound-color synesthesia (Zamm et al., 2013). Although these lines of evidence do not provide evidence of a causal connection among developmental synesthesia, autism and savant syndrome, results are suggestive that local hyperconnectivity is a common feature of these conditions.

However, we do not yet have enough data to draw any firm conclusions about the role of serotonin in developmental synesthesia in non-autistic individuals or the connection between developmental synesthesia and autism. More systematic population studies as well as whole-genome linkage scans and family linkage analyses may be able to shed more light on this connection. The extent to which serotonin is mechanistically involved in developmental synesthesia could be tested more systematically in pharmacological studies using drugs known to inhibit 5-HT2A receptors, such as SSRIs (Brang and Ramachandran, 2007), and serotonin-agonists, such as cocaine, that are only reported to cause hallucinations with excessive use. If serotonin is functionally implicated in synesthesia, we should expect 5-HT2A inhibitors to block or reduce synesthesia and serotonin-agonists to augment the experiences. A negative result would rule out that serotonin is functionally involved in synesthesia but would leave open the possibility that the neurotransmitter is involved in the onset of developmental synesthesia by leading to an early change in structural connectivity.

## CONCLUSION

The primary aim here has been to defend the hypothesis that synesthesia is at least sometimes a hyperserotonergic condition that triggers synesthesia through excitatory neurotransmitter action. The main evidence in favor of this hypothesis can be summarized as follows:

First of all, brain injury that gives rise to acquired synesthesia leads to necrosis and excessive release of serotonin and glutamate. Though this increase in excitatory neurotransmitter activity leads to lower excitability in local areas through negative feedback within weeks, decreased activity in affected neural regions could lead to a disinhibitory enhancement of neural activity and connectivity in unaffected cortical regions. Alternatively, the early increase in serotonin levels could lead to the formation of unusual feature binding.

Secondly, administration of certain hallucinogenic serotonin agonists (e.g., psilocybin) induces synesthesia. The mechanism underlying drug-induced synesthesia plausibly involves serotonergic excitatory activity in layer V pyramidal neurons implicated in multisensory binding.

Thirdly, serotonin synthesis is typically increased unilaterally in individuals with autism, about 15 percent of which are believed to experience synesthesia compared to about 4 percent population-wide. In terms of a potential mechanism, one possibility is that serotonin causes aberrant structural binding in the spared neural regions that are also responsible for the savant skills found in 10 percent of autistic individuals.

## Conflict of Interest Statement

The author declares that the research was conducted in the absence of any commercial or financial relationships that could be construed as a potential conflict of interest.

## References

[B1] Abraham H. D. (1983). Visual phenomenology of the LSD flashback. *Arch. Gen. Psychiatry* 40 884–889 10.1001/archpsyc.1983.01790070074009 6135405

[B2] Afra P. Funke M. Matuso F. (2009). Acquired auditory–visual synesthesia: a window to early cross-modal sensory interactions. *Psychol. Res. Behav. Manag.* 2 31–37 10.2147/PRBM.S4481 22110319 PMC3218766

[B3] Aghajanian G. K. Marek G. J. (1999). Serotonin induces excitatory postsynaptic potentials in apical dendrites of neocortical pyramidal cells. *Neuropharmacology* 36 589–599 10.1016/S0028-3908(97)00051-8 9225284

[B4] Alstadhaug K. B. Benjaminsen E. (2010). Synesthesia and migraine: case report. *BMC Neurol.* 10:121 10.1186/1471-2377-10-121 PMC3004877 21138558

[B5] Armel K. C. Ramachandran V. S. (1999). Acquired synesthesia in Retinitis Pigmentosa. *Neurocase* 5 293–296 10.1080/13554799908411982

[B6] Asher J. E. Lamb J. A. Brocklebank D. Cazier J. B. Maestrini E. Addis L. (2009). A whole-genome scan and fine-mapping linkage study of auditory-visual synesthesia reveals evidence of linkage to chromosomes 2q24, 5q33, 6p12, and 12p12. *Am. J. Hum. Genet.* 84 1–7 10.1016/j.ajhg.2009.01.012 PMC2668015 19200526

[B7] Banissy M. J. Walsh V. Ward J. (2009). Enhanced sensory perception in synaesthesia. *Exp. Brain Res.* 196 565–571 10.1007/s00221-009-1888-0 19533108

[B8] Barkai E. Hasselmo M. E. (1994). Modulation of the input/output function of rat piriform cortex pyramidal cells. *J. Neurophysiol.* 72 644–658 7983526 10.1152/jn.1994.72.2.644

[B9] Baron-Cohen S. Ashwin E. Ashwin C. Tavassoli T. Chakrabarti B. (2009). Talent in autism: hyper-systemizing, hyper-attention to detail and sensory hypersensitivity. *Philos. Trans. R. Soc. Lond. B Biol. Sci.* 364 1377–1383 10.1098/rstb.2008.0337 19528020 PMC2677592

[B10] Baron-Cohen S. Bor D. Billington J. Asher J. E. Wheelwright S. Ashwin C. (2007). Savant memory in a man with colour form-number synaesthesia and Asperger syndrome. *J. Conscious. Stud.* 14 237–251

[B11] Baron-Cohen S. Burt L. Smith-Laittan F. Harrison J. Bolton P. (1996). Synaesthesia: prevalence and familiality. *Perception* 25 1073–1079 10.1068/p251073 8983047

[B12] Baron-Cohen S. Wyke M. Binnie C. (1987). Hearing words and seeing colors: an experimental investigation of synesthesia. *Perception* 16 761–767 10.1068/p160761 3454433

[B13] Beauchamp M. S. Ro T. (2008). Neural substrates of sound-touch synesthesia following a thalamic lesion. *J. Neurosci.* 28 13696–13702 10.1523/JNEUROSCI.3872-08.2008 19074042 PMC6671766

[B14] Behrendt R. P. Young C. (2004). Hallucinations in schizophrenia, sensory impairment, and brain disease: a unifying model. *Behav. Brain Sci.* 27 771–787 10.1017/S0140525X04000184 16035402

[B15] Bor D. Billington J. Baron-Cohen S. (2007). Savant memory for digits in a case of synaesthesia and Asperger syndrome is related to hyperactivity in the lateral prefrontal cortex. *Neurocase* 13 311–319 10.1080/13554790701844945 18781431

[B16] Brang D. Ramachandran V. S. (2007). Psychopharmacology of synesthesia; the role of serotonin S2a receptor activation. *Med. Hypotheses* 70 903–904 10.1016/j.mehy.2007.09.007 17980498

[B17] Brang D. Ramachandran V. S. (2011). Survival of the synesthesia gene: why do people hear colors and taste words? *PLoS Biol*. 9:e1001205 10.1371/journal.pbio.1001205 PMC3222625 22131906

[B18] Brogaard B. (2012). “Color synesthesia,” in *Cognition and Language, Encyclopedia of Color Science and Technology* ed. Jameson K. A. (Berlin: Springer), (in press)

[B19] Brogaard B. (2013). “Synesthetic binding and the reactivation model of memory,” in *Sensory Blendings: New Essays on Synaesthesia* Deroy O. Nudds M. (Oxford: Oxford University Press), (in press)

[B20] Brogaard B. Marlow K. (2013). From brain damage to beethoven. How a head injury created a musical prodigy. *Guru Mag.* 11 10–14

[B21] Brogaard B. Marlow K. Rice K. (2013). “The long-term potentiation model for grapheme-color binding in synesthesia,” in Sensory Integration and the Unity of Consciousness eds Bennett D. Hill C. (Cambridge: MIT Press), (in press)

[B22] Brogaard B. Vanni S. Silvanto J. (2012). Seeing mathematics: perceptual experience and brain activity in acquired synesthesia. *Neurocase* 10.1080/13554794.2012.701646 [Epub ahead of print] 22937821

[B23] Bullock R. Zauner A. Woodward J. J. Myseros J. Choi S. C. Ward J. D. (1998). Factors affecting excitatory amino acid release following severe human head injury. *J. Neurosurg.* 89 507–518 10.3171/jns.1998.89.4.0507 9761042

[B24] Busto R. Dietrich W. D. Globus M. Y. Alonso O. Ginsberg M. D. (1997). Extracellular release of serotonin following fluid-percussion brain injury in rats. *J. Neurotrauma* 14 35–42 10.1089/neu.1997.14.35 9048309

[B25] Carhart-Harris R. L. Erritzoe D. Williams T. Stone J. M. Reed L. J. Colasanti A. (2012). Neural correlates of the psychedelic state as determined by fMRI studies with psilocybin. *Proc. Natl. Acad. Sci. U.S.A.* 109 2138–2143 10.1073/pnas.1119598109 22308440 PMC3277566

[B26] Ceglia I. Carli M. Baviera M. Renoldi G. Calcagno E. Invernizzi R. W. (2004). The 5-HT receptor antagonist M100, 907 prevents extracellular glutamate rising in response to NMDA receptor blockade in the mPFC. *J. Neurochem.* 91 189–199 10.1111/j.1471-4159.2004.02704.x 15379899

[B27] Chandana S. R. Behen M. E. Juhasz C. Muzik O. Rothermel R. D. Mangner T. J. (2005). Significance of abnormalities in develop-mental trajectory and asymmetry of cortical serotonin synthesis in autism. *Int. J. Dev. Neurosci.* 23 171–182 10.1016/j.ijdevneu.2004.08.002 15749243

[B28] Chugani D. C. Muzik O. Behen M. Rothermel R. Janisse J. J. Lee J. (1999). Developmental changes in brain serotonin synthesis capacity in autistic and nonautistic children. *Ann. Neurol.* 45 287–295 10.1002/1531-8249(199903)45:3<287::AID-ANA3>3.0.CO;2-9 10072042

[B29] Chugani D. C. Muzik O. Rothermel R. Behen M. Chakraborty P. Mangner T. (1997). Altered serotonin synthesis in the dentatothalamocortical pathway in autistic boys. *Ann. Neurol.* 42 666–669 10.1002/ana.410420420 9382481

[B30] Ciranna L. (2006). Serotonin as a modulator of glutamate- and GABA-mediated neurotransmission: implications in physiological functions and in pathology. *Curr. Neuropharmacol.* 4 101–114 10.2174/157015906776359540 18615128 PMC2430669

[B31] Cohen Kadosh R. Henik A. Catena A. Walsh V. Fuentes L. J. (2009). Induced cross-modal synaesthetic experience without abnormal neuronal connections. *Psychol. Sci.* 20 258–265 10.1111/j.1467-9280.2009.02286.x 19175754

[B32] Cott C. Rock A. (2008). Phenomenology of N,N-dimethyl-tryptamine use: a thematic analysis. *J. Sci. Explor.* 22 359–370

[B33] Cytowic R. E. (1989). *Synesthesia: A Union of the Senses*. New York: Springer Verlag 10.1007/978-1-4612-3542-2

[B34] Cytowic R. E. (1997). “Synaesthesia: phenomenology and neuropsychology – a review of current knowledge,” in *Synaesthesia: Classic and Contemporary Readings* eds Baron-Cohen S. Harrison J. E. (Oxford: Blackwell) 17–39

[B35] DeLong G. R. (1999). Autism: new data suggest a new hypothesis. *Neurology* 52 911–916 10.1212/WNL.52.5.911 10102405

[B36] Devinsky O. Luciano D. (1991). Psychic phenomena in partial seizures. *Sem. Neurol.* 11 100–109 10.1055/s-2008-1041211 1925125

[B37] Dixon M. J. Smilek D. Duffy P. L. Zanna M. P. Merikle P. M. (2006). The role of meaning in grapheme-colour synaesthesia. *Cortex* 42 243–252 10.1016/S0010-9452(08)70349-6 16683498

[B38] Dixon M. J. Smilek D. Merikle P. M. (2004). Not all synaesthetes are created equal: projector versus associator synaesthetes. *Cogn. Affect. Behav. Neurosci.* 4 335–343 10.3758/CABN.4.3.335 15535169

[B39] Domino G. (1989). Synaesthesia and creativity in fine arts students: an empirical look. *Creat. Res. J.* 2 17–29 10.1080/10400418909534297

[B40] Dorman C. (1991). Exceptional calendar calculating ability after early left hemispherectomy. *Brain Cogn.* 15 26–36 10.1016/0278-2626(91)90013-X 2009172

[B41] Dovern A. Fink G. R. Fromme A. C. Wohlschläger A. M. Weiss P. H. Riedl V. (2012). Intrinsic network connectivity reflects consistency of synesthetic experiences. *J. Neurosci.* 32 7614–7621 10.1523/JNEUROSCI.5401-11.2012 22649240 PMC6703581

[B42] Eagleman D. M. Kagan A. D. Nelson S. S. Sagaram D. Sarma A. K. (2007). A standardized test battery for the study of Synesthesia. *J. Neurosci. Methods* 159 139–145 10.1016/j.jneumeth.2006.07.012 16919755 PMC4118597

[B43] Esterman M. Verstynen T. Ivry R. B. Robertson L. C. (2006). Coming unbound: disrupting automatic integration of synesthetic color and graphemes by transcranial magnetic stimulation of the right parietal lobe. *J. Cogn. Neurosci.* 18 1570–1576 10.1162/jocn.2006.18.9.1570 16989556

[B44] Eysel U. T. Schweigart G. Mittmann T. Eyding D. Qu Y. Vandesande F. (1999). Reorganization in the visual cortex after retinal and cortical damage. *Restor. Neurol. Neurosci.* 15 153–164 12671230

[B45] Fay L. (1987). That smarts! Accident leaves man with unforgettable gift. *Virginia Pilot* 31–33

[B46] Ffytche D. H. (2007). Visual hallucinatory syndromes: past, present, and future. *Dialog. Clin. Neurosci.* 9 173–189 10.31887/DCNS.2007.9.2/dffytche PMC3181850 17726916

[B47] Friedrichs H. (2009). *Die Psychologie des Meskalinrausches*. Berlin: Verlag für Wissenschaft und Bildung

[B48] Giza C. C. Prins M. L. (2006). Is being plastic fantastic? Mechanisms of altered plasticity after developmental traumatic brain injury. *Dev. Neurosci.* 28 364–379 10.1159/000094163 16943660 PMC4297630

[B49] Glennon R. A. (1990). Do classical hallucinogens act as 5-HT2 agonists or antagonists? *Neuropsychopharmacology* 3 509–517 2078284

[B50] González-Maeso J. Ang R. L. Yuen T. Chan P. Weisstaub N. V. López-Giménez J. F. (2008). Identification of a serotonin/glutamate receptor complex implicated in psychosis. *Nature* 452 93–97 10.1038/nature06612 18297054 PMC2743172

[B51] González-Maeso J. Weisstaub N. V. Zhou M. Chan P. Ivic L. Ang R. (2007). Hallucinogens recruit specific cortical 5-HT2A receptor-mediated signaling pathways to affect behavior. *Neuron* 53 439–452 10.1016/j.neuron.2007.01.008 17270739

[B52] Grossenbacher P. G. Lovelace C. T. (2001). Mechanisms of synesthesia: cognitive and physiological constraints. *Trends Cogn. Sci.* 5 36–41 10.1016/S1364-6613(00)01571-0 11164734

[B53] Grandin T. (1995). *Thinking in Pictures: My life with Autism*. New York: Doubleday

[B54] Guillery R. W. Harting J. K. (2003). Structure and connections of the thalamic reticular nucleus: advancing views over half a century. *J. Comp. Neurol.* 463 360-71 10.1002/cne.10738 12836172

[B55] Hanggi J. Wotruba D. Jäncke L. (2011). Globally altered structural brain network topology in grapheme-color synesthesia. *J. Neurosci.* 31 5816–5828 10.1523/JNEUROSCI.0964-10.2011 21490223 PMC6622814

[B56] Harrison J. Hare D. J. (2004). Brief report: assessment of sensory abnormalities in people with autistic spectrum disorders. *J. Autism Dev. Disord.* 34 727–730 10.1007/s10803-004-5293-z 15679192

[B57] Hier D. B. LeMay M. Rosenberger P. B. (1979). Autism and unfavorable left-right asymmetries of the brain. *J. Autism Dev. Disord.* 9 153–159 10.1007/BF01531531 479099

[B58] Hintzen A. Passie T. (2010). *The Pharmacology of LSD*. Oxford, New York: Oxford University Press

[B59] Hinzman J. M. Thomas T. C. Burmeister J. J. Quintero J. E. Huettl P. Pomerleau F. (2010). Diffuse brain injury elevates tonic glutamate levels and potassium-evoked glutamate release in discrete brain regions at two days post-injury: an enzyme-based microelectrode array study. *J. Neurotrauma* 27 889–899 10.1089/neu.2009.1238 20233041 PMC2943939

[B60] Hubbard E. M. (2007). Neurophysiology of synesthesia. *Curr. Psychiatry Rep.* 9 193–199 10.1007/s11920-007-0018-6 17521514

[B61] Hubbard E. M. Manohar S. Ramachandran V. S. (2005). Contrast affects the strength of synesthetic colors. *Cortex* 184–194 10.1016/s0010-9452(08)70343-5 16683492

[B62] Jacobs L. Karpik A. Bozian D. (1981). Auditory–visual synesthesia: sound-induced photism. *Arch. Neurol.* 38 211–216 10.1001/archneur.1981.00510040037005 7213144

[B63] Jacome E. Gumnit R. J. (1979). Audioalgesic and audiovisuoalgesic synesthesias: epileptic manifestation. *Neurology* 29 1050–1053 10.1212/WNL.29.7.1050 572935

[B64] Jancke L. Beeli G. Eulig C. Hanggi J. (2009). The neuroanatomy of grapheme-color synesthesia. *Eur. J. Neurosci.* 29 1287–1293 10.1111/j.1460-9568.2009.06673.x 19302164

[B65] Kamal S. M. (2010). Pharmacological modulation of brain levels of glutamate and GABA in rats exposed to total sleep deprivation. *J. Exp. Pharmacol.* 2 65–71 10.2147/JEP.S11143 PMC4863290 27186093

[B66] Kemner C. Verbaten M. N. Cuperus J. M. Camfferman G van Engeland H. (1995). Auditory event-related brain potentials in autistic children and three different control groups. *Biol. Psychiatry* 38 150–165 10.1016/0006-3223(94)00247-Z 7578658

[B67] Kim U. McCormick D. A. (1998). The functional influence of burst and tonic firing mode on synaptic interactions in the thalamus. *J. Neurosci.* 18 9500–9516 9801387 10.1523/JNEUROSCI.18-22-09500.1998 PMC6792899

[B68] Kometer M. Schmidt A. Jäncke L. Vollenweider F. X. (2013). Activation of serotonin 2A receptors underlies the psilocybin-induced effects on oscillations, N170 visual-evoked potentials, and visual hallucinations. *J. Neurosci.* 33 10544–10551 10.1523/JNEUROSCI.3007-12.2013 23785166 PMC6618596

[B69] Kramer K. Azmitia E. C. Whitaker-Azmitia P. M. (1994). In vitro release of [3H]5-hydroxytryptamine from fetal and maternal brain by drugs of abuse. *Brain Res. Dev.* 78 142–146 10.1016/0165-3806(94)90019-1 7911745

[B70] Klimesch W. (2011). Evoked and early access to the knowledge system: the P1 inhibition timing hypothesis. *Brain Res.* 1408 52–71 10.1016/j.brainres.2011.06.003 21774917 PMC3158852

[B71] Konrad C. Geburek A. J. Rist F. Blumenroth H. Fischer B. Husstedt I. (2011). Long-term cognitive and emotional consequences of mild traumatic brain injury. *Psychol. Med.* 41 1197–1211 10.1017/S0033291710001728 20860865

[B72] Leboyer M. Philippe A. Bouvard M. Guilloud-Bataille M. Bondoux D. Tabuteau F. (1999). Whole blood serotonin and plasma beta-endorphin in autistic probands and their first-degree relatives. *Biol. Psychiatry* 45 158–163 10.1016/S0006-3223(97)00532-5 9951562

[B73] Lee H.-M. Roth B. L. (2012). Hallucinogen actions on human brain revealed. *Proc. Natl. Acad. Sci. U.S.A.* 109 1820–1821 10.1073/pnas.1121358109 22308478 PMC3277578

[B74] Lessell S. Cohen M. M. (1979). Phosphenes induced by sound. *Neurology* 29 1524–1526 10.1212/WNL.29.11.1524 574208

[B75] Loui P. Li H. C. Hohmann A. Schlaug G. (2011). Enhanced cortical connectivity in absolute pitch musicians: a model for local hyperconnectivity. *J. Cogn. Neurosci.* 23 1015–1026 10.1162/jocn.2010.21500 20515408 PMC3012137

[B76] Lythgoe M. Pollak T. Kalmas M. de Hann M. Chong W. K. (2005). Obsessive, prolific artistic output following subarachnoid hemorrhage. *Neurology* 64 397–398 10.1212/01.WNL.0000150526.09499.3E 15668459

[B77] Markram H. Toledo-Rodriguez M. Wang Y. Gupta A. Silberberg G. Wu C. (2004). Interneurons of the neocortical inhibitory system. *Nat. Rev. Neurosci.* 5 793–807 10.1038/nrn1519 15378039

[B78] Mattingley J. B. Rich A. N. Yelland G. Bradshaw J. L. (2001). Unconscious priming eliminates automatic binding of colour and alphanumeric form in synaesthesia. *Nature* 410 580–582 10.1038/35069062 11279495

[B79] McDougle C. J. Naylor S. T. Cohen D. J. Aghajanian G. K. Heninger G. R. Price L. H. (1996). Effects of tryptophan depletion in drug-free adults with autistic disorder. *Arch. Gen. Psychiatry* 53 993–1000 10.1001/archpsyc.1996.01830110029004 8911222

[B80] Miller B. Boon K. Cummings J. L. Read S. L. Mishkin F. (2000). Functional correlates of musical and visual ability in frontotemporal dementia. *Br. J. Psychiatry* 176 458–463 10.1192/bjp.176.5.458 10912222

[B81] Miller B. L. Cummings J. Mishkin F. Boone K. Prince F. Ponton M. (1998). Emergence of artistic talent in fronto-temporal dementia. *Neurology* 51 978–982 10.1212/WNL.51.4.978 9781516

[B82] Milner B. (1973). “Hemispheric specialization: scope and limits,” in *The Neurosciences: Third Study Program* eds Schmidt F. O. Worden F. G. (Cambridge: MIT Press) 75–89

[B83] Mueller S. Keeser D. Samson A. C. Kirsch V. Blautzik J. Grothe M. (2013). Convergent findings of altered functional and structural brain connectivity in individuals with high functioning autism: a multimodal MRI study. *PLoS ONE* 8:e67329 10.1371/journal.pone.0067329 PMC3688993 23825652

[B84] Mulvenna C. Walsh V. (2005). Synaesthesia. *Curr. Biol.* 15 R399–R400 10.1016/j.cub.2005.05.039 15936255

[B85] Neufeld J. Sinke C. Zedler M. Dillo W. Emrich H. M. Bleich S. (2012). Disinhibited feedback as a cause of synesthesia: evidence from a functional connectivity study on auditory-visual synesthetes. *Neuropsychologia* 50 1471–1477 10.1016/j.neuropsychologia.2012.02.032 22414594

[B86] Newbury D. F. Warburton P. C. Wilson N. Bacchelli E. Carone S. Lamb J. A. (2009). Mapping of partially overlapping de novo deletions across an autism susceptibility region (AUTS5) in two unrelated individuals affected by developmental delays with communication impairment. *Am. J. Med. Genet. A* 149A 588–597 10.1002/ajmg.a.32704 19267418 PMC2680219

[B87] Nichols D. E. (2004). Hallucinogens. *Pharmacol. Ther.* 101 131–181 10.1016/j.pharmthera.2003.11.002 14761703

[B88] Ornitz E. M. Guthrie., D., Farley A. J. (1978). “The early symptoms of childhood autism,” in *Cognitive Defects in the Development of Mental Illness* ed. Serban G. (New York: Brunner/Mazel) 24–42

[B89] Perez-Polo J. R. Rea H. C. Johnson K. M. Parsley M. A. Unabia G. C. Xu G. (2013). Inflammatory consequences in a rodent model of mild traumatic brain injury. *J. Neurotrauma* 30 727–740 10.1089/neu.2012.2650 23360201 PMC3941841

[B90] Pesenti M. Zago L. Crivello F. (2001). Mental calculation in a prodigy is sustained by right prefrontal and medial temporal areas. *Nat. Neurosci.* 4 103–107 10.1038/82831 11135652

[B91] Pralong E. Magistretti P. Stoop R. (2002). Cellular perspectives on the glutamate-monoamine interactions in limbic lobe structures and their relevance for some psychiatric disorders. *Prog. Neurobiol.* 67 173–202 10.1016/S0301-0082(02)00017-5 12169296

[B92] Presti D. Nichols D. (2004). “Biochemistry and neuropharmacology of psilocybin mushrooms,” in *Teonanacatl: Sacred Mushroom of Vision* ed. Metzner R. (El Verano, CA: Four Trees) 89–108

[B93] Previc F. (2011). “Dopamine, altered consciousness, and distant space with special reference to shamanic ecstasy,” in *Altering Consciousness: A Multidisciplinary Perspective Vol. 2. Biological and Psychological Perspectives* eds Cardeña E. Winkelman M. (Westport, CT: Praeger) 43–61

[B94] Ramachandran V. S. Hubbard E. M. (2001a). Psychophysical investigations into the neural basis of synaesthesia. *Proc. R. Soc. B Biol. Sci.* 268 979–983 10.1098/rspb.2001.1576 PMC1088697 11370973

[B95] Ramachandran V. S. Hubbard E. M. (2001b). Synaesthesia: a window into perception, thought and language. *J. Conscious. Stud.* 8 3–34

[B96] Rich A. N. Mattingley J. B. (2002). Anomalous perception in synaesthesia: a cognitive neuroscience perspective. *Nat. Rev. Neurosci.* 3 43–52 10.1038/nrn702 11823804

[B97] Ro T. Ellmore T. M. Beauchamp M. S. (2012). A neural link between feeling and hearing. *Cereb. Cortex* 23 1724–1730 10.1093/cercor/bhs166 22693344 PMC3673182

[B98] Ro T. Farne A. Johnson R. M. Weeden V. Chu Z. Wang Z. J. (2007). Feeling sound after a thalamic lesion. *Ann. Neurol.* 62 433–441 10.1002/ana.21219 17893864

[B99] Robertson C. S. Bell M. J. Kochanek P. M. Adelson P. D. Ruppel R. A. Carcillo J. A. (2001). Increased adenosine in cerebrospinal fluid after severe traumatic brain injury in infants and children: association with severity of injury and excitotoxicity. *Crit. Care Med.* 29 2287–3393 10.1097/00003246-200112000-00009 11801827

[B100] Rogowska A. (2011). Categorization of synaesthesia. *Rev. Gen. Psychol.* 15 213–227 10.1037/a0024078

[B101] Rothen N. Meier B. (2010). Higher prevalence of synaesthesia in art students. *Perception* 39 718–720 10.1068/p6680 20677709

[B102] Rothen N. Meier B. Ward J. (2012). Enhanced memory ability: insights from synaesthesia. *Neurosci. Biobehav. Rev.* 36 1952–1963 10.1016/j.neubiorev.2012.05.004 22634573

[B103] Rouw R. Scholte H. S. (2007). Increased structural connectivity in grapheme-color synesthesia. *Nat. Neurosci.* 10 792–797 10.1038/nn1906 17515901

[B104] Sacks O. (2012). *Hallucinations*. Toronto: Alfred A. Knopf

[B105] Sagiv N. Ilbeigi A. Ben-Tal O. (2011). Reflections on synesthesia, perception, and cognition. *Intellectica* 55 81–94

[B106] Sagiv N. Ward J. (2006). Crossmodal interactions: lessons from synesthesia. *Prog. Brain Res.* 155 259–271 10.1016/S0079-6123(06)55015-0 17027393

[B107] Schain R. J. Freedman D. X. (1961). Studies on 5-hydroxyindole metabolism in autistic and other mentally retarded children. *J. Pediatr.* 58 315–320 10.1016/S0022-3476(61)80261-8 13747230

[B108] Schott G. D. (2012). Pictures as a neurological tool: lessons from enhanced and emergent artistry in brain disease. *Brain* 135 1947–1963 10.1093/brain/awr314 22300875

[B109] Schroeder C. E. Foxe J. (2005). Multisensory contributions to low-level, ‘unisensory’ processing. *Curr. Opin. Neurobiol.* 15 454–458 10.1016/j.conb.2005.06.008 16019202

[B110] Scruggs J. L. Schmidt D. Deutch A. Y. (2003). The hallucinogen 1-[2,5-dimethoxy-4-iodophenyl]-2-aminopropane (DOI) increases cortical extracellular glutamate levels in rats. *Neurosci. Lett.* 346 137–140 10.1016/S0304-3940(03)00547-0 12853103

[B111] Shanon B. (2002). *The Antipodes of the Mind*. Oxford, NY: Oxford University Press

[B112] Shanon B. (2003). Three stories concerning synaesthesia – a commentary on the paper by Ramachandran and Hubbard. *J. Conscious. Studies* 10 69–74

[B113] Simner J. Mulvenna C. Sagiv N. Tsakanikos E. Witherby S. A. Fraser C. (2006). Synaesthesia: the prevalence of atypical cross-modal experiences. *Perception* 35 1024–1033 10.1068/p5469 17076063

[B114] Sinke C. Halpern J. H. Zedler M. Neufeld J. Emrich H. M. Passie T. (2012). Genuine and drug-induced synesthesia: a comparison. *Conscious. Cogn.* 21 1419–1434 10.1016/j.concog.2012.03.009 22521474

[B115] Snyder A. (2009). Explaining and inducing savant skills: privileged access to lower level, less-processed information. *Philos. Trans. R. Soc. Lond. B Biol. Sci.* 364 1399–1405 10.1098/rstb.2008.0290 19528023 PMC2677578

[B116] Snyder A. W. Mulcahy E. Taylor J. L. Mitchell D. Sachdev P. Gandevia S. C. (2003). Savant-like skills exposed in normal people by suppressing the left fronto-temporal lobe. *J. Integr. Neurosci.* 2 149–158 10.1142/S0219635203000287 15011267

[B117] Takeuchi N. Oouchida Y. Izumi S.-I. (2012). Motor control and neural plasticity through interhemispheric interactions. *Neural Plast.* 2012 823285 10.1155/2012/823285 PMC3541646 23326685

[B118] Tammet D. (2006). *Born on a Blue Day. Inside the Extraordinary Mind of an Autistic Savant*. New York: Free Press

[B119] Terhune D. B Cohen Kadosh R. (2012). “Synaesthesias,” in *Hallucinations: Research and Practice* eds Blom J. D. Sommer I. E. C. (New York: Springer) 91–104 10.1007/978-1-4614-0959-5_7

[B120] Terhune D. B. Tai S. Cowey A. Popescu T Cohen Kadosh R. (2011). Enhanced cortical excitability in grapheme-color synesthesia and its modulation. *Curr. Biol.* 21 2006–2009 10.1016/j.cub.2011.10.032 22100060 PMC3242051

[B121] Thomas-Anterion C. Creac’h C. Dionet E. Borg C. Extier C. Faillenot I. (2010). De novo artistic activity following insular-SII ischemia. *Pain* 150 121–127 10.1016/j.pain.2010.04.010 20447767

[B122] Tomson S. N. Avidan N. Lee K. Sarma A. K. Tushe R. Milewicz D. M. (2011). The genetics of colored sequence synesthesia: suggestive evidence of linkage to 16q and genetic heterogeneity for the condition. *Behav. Brain Res.* 223 48–52 10.1016/j.bbr.2011.03.071 21504763 PMC4075137

[B123] Torres-Escalante J. L. Barral J. A. Ibarra-Villa M. D. Perez-Burgos A. Gongora-Alfaro J. L. Pineda J. C. (2004). 5-HT1A, 5-HT2, and GABAB receptors interact to modulate neurotransmitter release probability in layer 2/3 somatosensory rat cortex as evaluated by the paired pulse protocol. *J. Neurosci. Res.* 78 268–278 10.1002/jnr.20247 15378508

[B124] Treffert D. A. (2006). *Extraordinary People: Understanding Savant Syndrome*. New York: Ballantine Books

[B125] Treffert D. A. (2009). The savant syndrome: an extraordinary condition. A synopsis: past, present, future. *Philos. Trans. R. Soc. B Biol. Sci.* 27 1351–1357 10.1098/rstb.2008.0326 PMC2677584 19528017

[B126] Trudeau L. E. (2004). Glutamate co-transmission as an emerging concept in monoamine neuron function. *J. Psychiatry Neurosci.* 29 296–310 15309046 PMC446224

[B127] Veenstra-VanderWeele J. Muller C. L. Iwamoto H. Sauer J. E. Owens W. A. Shah C. R. (2012). Autism gene variant causes hyperserotonemia, serotonin receptor hypersensitivity, social impairment and repetitive behavior. *Proc. Natl. Acad. Sci. U.S.A.* 109 5469–5474 10.1073/pnas.1112345109 22431635 PMC3325657

[B128] Vollenweider F. X. Leenders K. L. Scharfetter C. Maguire P. Stadelmann O. Angst J. (1997). Positron emission tomography and fluorodeoxyglucose studies of metabolic hyperfrontality and psychopathology in the psilocybin model of psychosis. *Neuropsychopharmacology* 16 357–372 10.1016/S0893-133X(96)00246-1 9109107

[B129] Vollenweider F. X. Vollenweider-Scherpenhuyzen M. F. I. Bäbler A. Vogel H. Hell D. (1998). Psilocybin induces schizophrenia-like psychosis in humans via a serotonin-2 agonist action. *NeuroReport* 9 3897–3902 10.1097/00001756-199812010-00024 9875725

[B130] Walsh R. (2005). Can synaesthesia be cultivated?: indications from surveys of meditators. *J. Conscious. Studies* 12 5–17

[B131] Ward J. (2013). Synesthesia. *Annu. Rev. Psychol.* 64 49–75 10.1146/annurev-psych-113011-143840 22747246

[B132] Ward J. Thompson-Lake D. Ely R. Kaminski F. (2008). Synaesthesia, creativity and art: what is the link? *Br. J. Psychol.* 99 127–141 10.1348/000712607X204164 17535472

[B133] Ward J. Wright T. (2012). Sensory substitution as an artificially acquired synaesthesia. *Neurosci. Biobehav. Rev.* 10.1016/j.neubiorev.2012.07.007 [Epub ahead of print] 22885223

[B134] Werner C. Engelhard K. (2007). Pathophysiology of traumatic brain injury. *Br. J. Anaesth.* 99 4–9 10.1093/bja/aem131 17573392

[B135] Whitaker-Azmitia P. M. (2005). Behavioral and cellular consequences of increasing serotonergic activity during brain development: a role in autism? *Int. J. Dev. Neurosci.* 23 75–83 10.1016/j.ijdevneu.2004.07.022 15730889

[B136] Whitaker-Azmitia P. M. Zhang X. Clarke C. (1994). Effects of gestational exposure to monoamine oxidase inhibitors in rats: preliminary behavioral and neurochemical studies. *Neuropsychopharmacology* 11 125–132 10.1038/npp.1994.42 7840864

[B137] Wiess P. H. Fink G. R. (2009). Grapheme-colour synaesthetes show increased grey matter volumes of parietal and fusiform cortex. *Brain* 132 65–70 10.1093/brain/awn304 19028762

[B138] Yaro C. Ward J. (2007). Searching for shereshevskii: what is superior about the memory of synaesthetes? *Q. J. Exp. Psychol. (Hove)* 60 681–695 10.1080/17470210600785208 17455076

[B139] Young R. L. Ridding M. C. Morrell T. L. (2004). Switching skills by turning off part of the brain. *Neurocase* 10 215–222 10.1080/13554790490495140 15788259

[B140] Yuan J. (2009). Neuroprotective strategies targeting apoptotic and necrotic cell death for stroke. *Apoptosis* 14 469–477 10.1007/s10495-008-0304-8 19137430 PMC2745337

[B141] Zamm A. Schlaug G. Eagleman D. M. Loui P. (2013). Pathways to seeing music: enhanced structural connectivity in colored-music synesthesia. *Neuroimage* 74 359–366 10.1016/j.neuroimage.2013.02.024 23454047 PMC3643691

